# Community-Acquired* Moraxella catarrhalis* Bacteremic Pneumonia: Two Case Reports and Review of the Literature

**DOI:** 10.1155/2016/5134969

**Published:** 2016-02-18

**Authors:** Miguel Angel Ariza-Prota, Ana Pando-Sandoval, Marta García-Clemente, David Fole-Vázquez, Pere Casan

**Affiliations:** Hospital Universitario Central de Asturias (HUCA), Instituto Nacional de Silicosis (INS), Área del Pulmón, Facultad de Medicina, Universidad de Oviedo, 33005 Oviedo, Spain

## Abstract

*Moraxella* (formerly* Branhamella*)* catarrhalis* was discovered at the end of the nineteenth century, and for many decades it was considered to be a harmless commensal of the upper respiratory tract. It is a Gram-negative, aerobic diplococcus considered to be the third most common pathogen isolated in childhood sinusitis and otitis media and in adult chronic lower respiratory disease, as well as an etiological agent of pneumonia in immunosuppressed patients or those with chronic obstructive pulmonary disease.* Moraxella catarrhalis* pneumonia is rarely associated with bacteremia. Here, we present two cases of community-acquired* Moraxella catarrhalis* bacteremic pneumonia.

## 1. Introduction

*Moraxella catarrhalis* is an important pathogen that causes a lower respiratory tract infection in healthy hosts and patients with chronic lung disease. Although it is a major pathogen of the lower respiratory tract, it rarely causes bacteremia [1]. Here, we present two cases of community-acquired* Moraxella catarrhalis* bacteremic pneumonia in adults; the first case is in an immunosuppressed patient, and the second is in a healthy immunocompetent patient.

## 2. Case Presentation 1

An 85-year-old Spanish female was admitted to our hospital in March 2015, after she had experienced five days with a cough, hemoptoic sputum, dyspnea, and a fever. She had no known toxic habits or surgical background. She was diagnosed with tuberculosis when she was a child, and in 2009,* Mycobacterium fortuitum* was isolated in a sputum culture for which she received treatment with Isoniazid, Rifampin, and Pyrazinamide. She was diagnosed with Diffuse Large B Cell Non-Hodgkin Lymphoma (stage 1A) in 2007 and received treatment with Rituximab and complementary radiotherapy with a favorable response. She was also diagnosed with cryoglobulinemic vasculitis in 1999 and was on corticosteroid treatment at the time.

Clinical findings were as follows: body temperature 36.2°C, blood pressure 96/60 mmHg, heart rate 160 beats/min, respiratory rate 30 breaths/min, and oxygen saturation 88% (room air). The physical examination revealed a disseminated petechial rash over her body; pulmonary auscultation showed diffuse rhonchi and diminished respiratory sounds at the left lung base. Laboratory tests revealed the following: 11.7 g hemoglobin; white blood cell count 5,000 × 10^9^/L with 90% neutrophils; C-reactive protein level 4.0 mg/dL; procalcitonin level 1.06 *μ*g/L; and the platelet count, arterial blood clotting, and the rest of the biochemical tests were within normal ranges.

**Figure 1:**
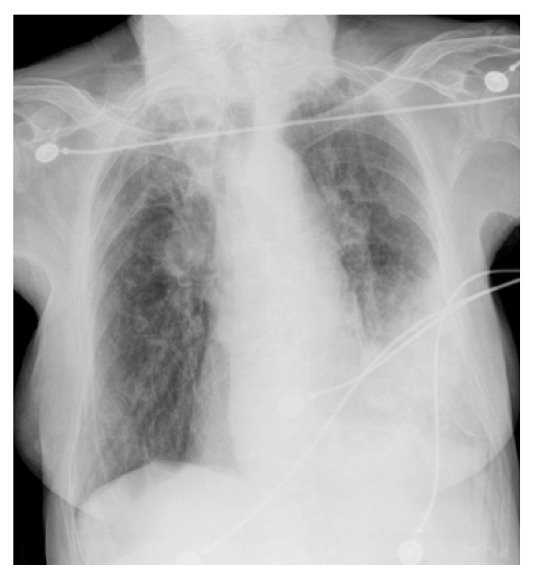
Chest X-ray on admission: left lower lobe alveolar infiltrate.

The chest X-ray revealed a left lung basal infiltrate (Figure 1). All three blood cultures obtained on admission showed* Moraxella catarrhalis* growth, which was* B-lactamase-positive* and susceptible to Ceftriaxone, Azithromycin, and Levofloxacin. Sputum cultures were negative, and a chest X-ray was performed during her hospitalization showing radiological improvement. The patient was treated with Levofloxacin for two weeks with clinical improvement and was discharged. She suffered a left femur fracture three months later and died during her hospitalization.

## 3. Case Presentation 2

A 76-year-old Spanish female was referred to our hospital in January 2015 because she had experienced three days with a fever and cough with nonproductive sputum. She had no known toxic habits or surgical or medical background. Clinical findings were as follows: body temperature 38°C, blood pressure 130/90 mmHg, heart rate 90 beats/min, respiratory rate 22 breaths/min, and oxygen saturation 92% (room air). The physical examination was normal, except for pulmonary auscultation where diminished respiratory sounds and crackles were found bilaterally at the bases of both lungs. Laboratory tests (complete blood count, biochemical and arterial blood clotting) were within normal ranges, except for the C-reactive protein level which was 4.2 mg/dL.

**Figure 2:**
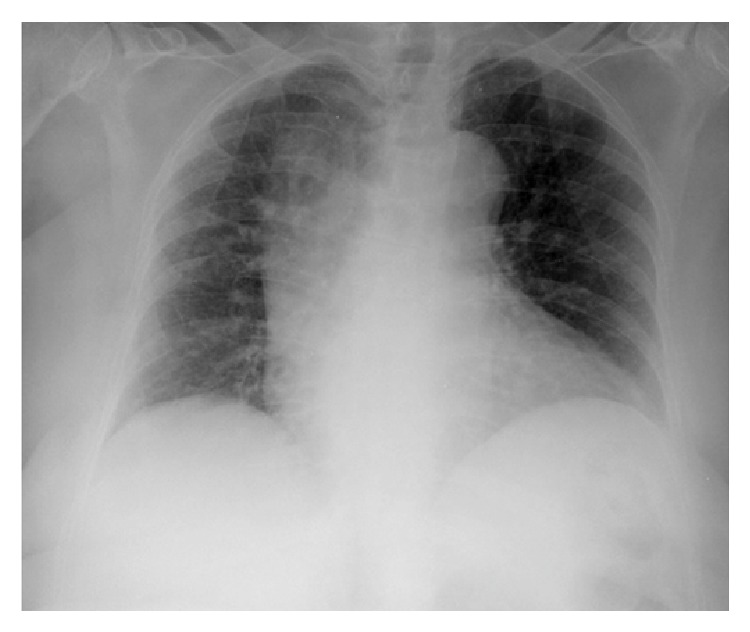
Chest X-ray on admission: right upper lobe alveolar infiltrate.

The chest X-ray showed an alveolar infiltrate in the right upper lobe (Figure 2). Blood cultures showed* Moraxella catarrhalis* growth (*B-lactamase negative*). A control chest X-ray showed radiological improvement. The patient was treated with amoxicillin/clavulanic acid for ten days with clinical improvement and was discharged. She remained well five months later.

## 4. Discussion

*Moraxella catarrhalis*, while it is a major pathogen of the lower respiratory tract, rarely causes bacteremia [1].* M. catarrhalis* is a Gram-negative, aerobic diplococcus, which has undergone several changes in nomenclature and periodic changes in its perceived status as either a commensal or a pathogen [2]. It is now accepted as the third most common pathogen of the respiratory tract after* Streptococcus pneumoniae* and* Haemophilus influenzae*. In immunocompromised hosts, the bacterium can cause a variety of severe infections, including pneumonia, endocarditis, septicemia, and meningitis [3].

In a report of 58 cases of* Moraxella catarrhalis* bacteremia, neutropenia was the leading underlying disorder [4]. Neutrophils are considered to be important for the host defense against* M. Catarrhalis* [4].* M. catarrhalis* bacteremia has nonspecific symptoms, being particularly severe and fatal in immunocompromised patients. Therefore, mortality may vary depending on comorbid illness and clinical presentation of the infection. Skin lesions such as purpuric and petechial rash were observed in our first case. Although there are no pathognomonic clinical signs suggesting* M. catarrhalis* bacteremia, it has been reported that petechiae and purpura are observed in 25% of patients, and most commonly in children [5].

We used PubMed (http://www.ncbi.nlm.nih.gov/PubMed/) to search for literature on* M. catarrhalis* bacteremic pneumonia with the following index words:* Moraxella (Branhamella) catarrhalis* bacteremic pneumonia. A total of 8 clinical cases were found in the search. Only one of these cases was an immunocompetent patient; the rest were immunocompromised hosts. In our second case, the patient was healthy and not immunosuppressed, which makes it even rarer and more interesting due to its almost zero frequency. In a report of* Moraxella* isolates submitted to the Centers for Disease Control and Prevention (CDC) in the United States between 1953 and 1980, no strains of* M. catarrhalis* were isolated from the blood [6]. It has been reported that 45% of* M. catarrhalis* pneumonia patients died of their underlying disease within three months of hospital admission [7]. The seasonal recovery of* M. catarrhalis* in respiratory infections is significantly increased during the late fall through early spring period [8]. This seasonal pattern raises the possibility of a viral “trigger” mechanism responsible for respiratory tract colonization or infection with* M. catarrhalis* [8]. One of the two isolates of our patients was* beta-lactamase* positive. B-lactamases, especially BRO-1, are found in 67% to 80% of* M. catarrhalis* strains [7].

Three multicenter studies (the Susceptibility to the Antimicrobials Used in the Community in Spain [SAUCE] studies) have been carried out to evaluate the antibiotic resistance rates among respiratory pathogens [9, 10]. One of the most remarkable findings was the increasing prevalence of isolates belonging to the *β*-lactamase-nonproducing ampicillin resistance (BLNAR) phenotype. However, neither the molecular basis of the Spanish BLNAR strains nor their molecular epidemiology has been characterized so far. Empiric use of penicillin, ampicillin, or amoxicillin for these organisms can no longer be recommended, and routine susceptibility testing is necessary. The* beta-lactamase* inhibitors clavulanic acid and sulbactam are highly active* in vitro* against* beta-lactamase* producing strains [11]. Although sporadic resistance to trimethoprim-sulfamethoxazole has been reported [12], this combination is also a good alternative. In the United States and Europe, the proportion of BLNAR isolates remains very low [13], while the proportion of BLNAR strains in Japan has rapidly increased over the last decade [14]. The high prevalence of these resistant organisms is attributed to the frequent use of oral and intravenous cephem antibiotic agents in Japan [15]. These BLNAR strains show higher levels of resistance to expanded- and broad-spectrum cephems than non-BLNAR strains, which can cause serious clinical problems.

## 5. Conclusion

We have reported two new cases of* Moraxella catarrhalis* bacteremic pneumonia. This is a very rare event, and only one case has been described in the literature regarding an immunocompetent patient. These cases illustrate not only the fact that* Moraxella* can cause bacteremia, but perhaps more importantly the fact that in diagnostic terms only the blood cultures allowed for a diagnosis. We believe that blood cultures should always be taken in cases of pneumonia and that the ability of this organism to produce* B-lactamase* and its potential to produce a fatal outcome should warn us that this is a pathogen that should not be underrated despite its low occurrence rate.
